# Novel functions of peroxiredoxin Q from *Deinococcus radiodurans* R1 as a peroxidase and a molecular chaperone

**DOI:** 10.1002/1873-3468.13302

**Published:** 2018-12-11

**Authors:** Chuloh Cho, Gun Woong Lee, Sung H. Hong, Shubhpreet Kaur, Kwang‐Woo Jung, Jong‐Hyun Jung, Sangyong Lim, Byung Yeoup Chung, Seung Sik Lee

**Affiliations:** ^1^ Advanced Radiation Technology Institute Korea Atomic Energy Research Institute Jeongeup Korea; ^2^ Jeonju AgroBio‐Materials Institute Korea; ^3^ Department of Radiation Biotechnology and Applied Radioisotope Korea University of Science and Technology Daejeon Korea

**Keywords:** *Deinococcus radiodurans* R1, DR0846, molecular chaperone, peroxidase, peroxiredoxin Q

## Abstract

*Deinococcus radiodurans* R1 is extremely resistant to ionizing radiation and oxidative stress. In this study, we characterized DR0846, a candidate peroxiredoxin in *D. radiodurans*. DR0846 is a peroxiredoxin Q containing two conserved cysteine residues. DR0846 exists mainly in monomeric form with an intramolecular disulfide bond between the two cysteine residues. We found that DR0846 functions as a molecular chaperone as well as a peroxidase. A mutational analysis indicates that the two cysteine residues are essential for enzymatic activity. A double‐deletion mutant lacking *DR0846* and catalase *DR1998* exhibits decreased oxidative and heat shock stress tolerance with respect to the single mutants or the wild‐type cells. These results suggest that DR0846 contributes to resistance against oxidative and heat stresses in *D. radiodurans*.

## Abbreviations

**AMS**, 4‐acetamido‐4′‐maleimidylstilbene‐2,2′‐disulfonic acid

**DSB**, DNA double‐strand break

**GR**, glutathione reductase

**GSH**, reduced glutathione

**H**_**2**_ **O**_**2**_, hydrogen peroxide

**HMW**, high molecular weight

**IR**, ionizing radiation

**MDH**, malate dehydrogenase

**NADH**, nicotinamide adenine dinucleotide

**PrxQ**, peroxiredoxin Q

**ROS**, reactive oxygen species

**SEC**, size exclusion chromatography

**SOD**, superoxide dismutases

**TCA**, trichloroacetic acid

**TF**, trigger factor

**TR**, thioredoxin reductase

**Trx**, thioredoxin

**WT**, wild‐type

*Deinococcus radiodurans* R1 is well‐known for its extreme resistance to ionizing radiation (IR) 1, 2, 3, 4. IR induces DNA double‐strand breaks and reactive oxygen species (ROS) in bacteria 3, 5, 6, 7. Although the IR resistance of *D. radiodurans* is due to its highly condensed nucleoid structure and enhanced DNA repair systems 3, some evidence suggests that it requires ROS scavenging systems 8, 9. *D. radiodurans* can remove ROS by nonenzymatic antioxidants, such as manganese complexes or carotenoids, and enzymatic antioxidants, such as catalases, superoxide dismutases (SOD), and peroxidases 3, 10. Catalases and peroxidases decompose hydrogen peroxide to water, whereas SOD converts superoxide radicals to hydrogen peroxide from the cells. *D. radiodurans* encodes two catalases (DR1998 and DRA0259), three SODs (DR1279, DR1546, and DRA0202), a cytochrome *c* peroxidase (DRA0301), an iron‐dependent peroxidase (DRA0145), and four peroxiredoxins (Prxs) (DR0846, DR1208, DR1209, and DR2242) 2, 3, 11.

Peroxiredoxins are a family of antioxidant enzymes that detoxifying hydrogen peroxide, alkylhydroperoxide, and peroxynitrite using thiols as electron donors 12, 13, 14. Prxs have highly conserved peroxidatic (*C*_P_) and resolving (*C*_R_) cysteine residues, which are essential for peroxidase activity 15, 16. Based on the absence or presence of conserved catalytic cysteine residues, Prxs are divided into three groups, typical 2‐Cys Prx, atypical 2‐Cys Prx, which are subdivided into type II Prx and PrxQ, and 1‐Cys Prx 17, 18. Typical and atypical 2‐Cys Prx have two Cys residues and 1‐Cys Prx lacks the resolving cysteine residue 17. PrxQ is a homolog of bacterioferritin co‐migratory protein (BCP) in *Escherichia coli* 19. PrxQ possesses two conserved cysteine residues (CXXXXC), with an intramolecular disulfide bond between the two cysteine residues, which is reduced by Trx 19. PrxQ usually functions as a monomeric peroxidase having high reactivity to H_2_O_2_ and butyl hydroperoxide 13. However, PrxQ4 (SsBcp4) in *Sulfolobus solfataricus* forms a non‐covalent dimeric structure and adopts an atypical 2‐Cys catalytic mechanism 20. Some PrxQ proteins function as peroxidases in a Grx‐dependent manner and only have a functional catalytic cysteine residue 21, 22. PrxQ plays an important role in *E. coli* and *Helicobacter pylori* under oxidative stress 21, 23. Despite many recent studies, the physiological functions, electron donors, and substrate specificity of PrxQs are largely unknown.

In this study, we characterized the expression and functions of DR0846 of *D. radiodurans*. We found that DR0846 is a PrxQ with two cysteine residues at positions 60 and 65. Additionally, we demonstrated that DR0846 exhibits a dual function as a peroxidase and a molecular chaperone *in vivo* and *in vitro*.

## Materials and methods

### Cell strains, growth conditions, and medium

*Deinococcus radiodurans* R1 (ATCC13939) was cultured in TGY broth (0.5% tryptone, 0.1% glucose, and 0.3% yeast extract) or on TGY plates at 30 °C. A stationary‐phase culture grown overnight with shaking was used as the seed culture. The seed culture was inoculated in TGY broth at a 1 : 100 dilution. *E. coli* DH5α (Promega, Madison, WI, USA) and BL21‐DE3 (Invitrogen, Carlsbad, CA, USA) strains were grown in Luria–Bertani (LB) broth (DB, Franklin Lakes, NJ, USA) or on LB plate at 37 °C. Antibiotics were used when necessary at the following concentrations: kanamycin (50 μg·mL^−1^) for *E. coli* and kanamycin (8 μg·mL^−1^) or chloramphenicol (3 μg·mL^−1^) for *D. radiodurans*.

### Bioinformatics analysis of peroxiredoxins

Analyses of the amino acid sequences, the isoelectric point, and molecular mass of proteins were performed using National Center for Biotechnology Information (https://www.ncbi.nlm.nih.gov/nucleotide/) and UCSC Archaea Genome Browser (http://archaea.ucsc.edu/lists/deinRadi/refSeq-list.html) databases. mega 7.0 was used to determine sequence identities and to build a phylogenetic tree using the neighbor‐joining method 24, 25, 26. Evolutionary distances were computed using the number of differences method 27 and are expressed as the number of amino acid differences between sequences. The analysis involved 34 amino acid sequences. All ambiguous positions were removed for each sequence pair.

### qRT‐PCR analysis of peroxiredoxin genes

For the qRT‐PCR analysis, cells were grown on TGY medium for 16 h at 30 °C. The seed culture was inoculated in TGY broth at a 1 : 100 dilution and grown at 30 °C until OD_600_ reached ~ 0.5 in TGY broth. For the oxidative stress treatment, cells were incubated for 5, 10, 20, or 30 min after treatment with 20 mm H_2_O_2_ at 30 °C. For the gamma ray treatment, cells were irradiated at 1, 3, or 5 kGy for 1 h. Total RNA was isolated from treated or untreated cells using TRI Reagent^®^ (Molecular Research Center, Inc., Cincinnati, OH, USA) according to the manufacturer's instructions.

### Cloning of *DR0846* and *DR0846* cysteine mutants

*DR0846* and its mutants were cloned in the pET‐28a (+) expression vector (Novagen, Madison, WI, USA). To construct *pET‐28a:DR0846*, the coding region was isolated from the genomic DNA of *D. radiodurans* R1 by PCR with primers harboring *Bam*HI (N terminus) and *Hin*dIII (C terminus) sites (Table S1) using *Pfu* DNA polymerase (Solgent, Gyeonggi‐do, Korea). The PCR products were inserted into the pGEM‐T Easy vector (Promega). The DNA fragments were cut with their corresponding restriction enzymes and cloned into the pET‐28a vector. The *pET‐28a:DR0846* plasmid was used as a template to generate the cysteine mutants C60S, C65S, and C60S/C65S by substituting Cys for Ser^60^, Ser^65^, and Ser^60, 65^ by PCR‐mediated site‐directed mutagenesis. All constructs were confirmed by DNA sequencing.

### Expression and purification of recombinant proteins

The *pET‐28a:DR0846*,* pET‐28a:DR0846C60S*,* pET‐28a:DR0846C65S*, and *pET‐28a:DR0846C60S/C65S* vectors were transformed into BL21‐DE3 and plated on LB plates containing 50 μg·mL^−1^ kanamycin. A single colony was inoculated in 5 mL of LB medium and grown overnight at 37 °C to obtain the seed culture. Then, the seed culture was inoculated in LB broth at a 1 : 100 dilution. The expression of His‐tagged DR0846 was induced with 0.1 mm isopropyl‐β‐d‐thiogalactopyranoside (IPTG) for 4 h at 30 °C. The recombinant proteins were purified using a nickel‐nitrilotriacetate‐agarose (Ni‐NTA) column (Peptron, Daejeon, Korea) following the manufacturer's instructions. The proteins were eluted by thrombin at 4 °C overnight. Purified proteins were dialyzed using 50 mm Tris/HCl (pH 7.5) for biochemical analyses.

### Size exclusion chromatography

Size exclusion chromatography (SEC) was performed at 25 °C to determine the size of DR0846 by fast protein liquid chromatography (AKTA, Amersham Biosciences, Uppsala, Sweden) using a Superdex 200 10/300 GL gel‐filtration column (Amersham Biosciences) following previously described methods, with minor modifications 28. The column was equilibrated and run with 50 mm Tris/HCl (pH 7.5) buffer at a flow rate of 0.5 mL·min^−1^ at 4 °C. Absorbance was monitored at 280 nm.

### Peroxidase activity assay

The peroxidase activity of purified DR0846 was measured by nicotinamide adenine dinucleotide phosphate (NADH) oxidation at 340 nm as described previously, with minor modifications 29. For thioredoxin‐dependent peroxidase activity, various concentrations of DR0846 were incubated with 0.3 mm NADH, 5 μm yeast thioredoxin reductase (TR), and 1 μm yeast thioredoxin (Trx) in 50 mm HEPES buffer (pH 8.0). For glutaredoxin‐dependent peroxidase activity, various concentrations of DR0846 were incubated with 0.3 mm NADH, 5 μm glutathione reductase (GR), and 1 mm reduced glutathione (GSH) in 50 mm Tris/HCl (pH 8.0), followed by the addition of 1 mm H_2_O_2_. NADH oxidation was monitored by measuring the change in absorbance at 340 nm for 10 min using a UV‐Visible spectrophotometer (Evolution 300 UV‐Vis Spectrophotometer; Thermo Scientific, Worcester, MA, USA).

### Molecular chaperone activity assay

Holdase chaperone activity was determined as described previously 29 by assessing the ability of recombinant DR0846 to inhibit the thermal aggregation of substrate proteins 30, 31, 32, 33. Briefly, malate dehydrogenase (MDH) was incubated in 50 mm HEPES buffer (pH 8.0) with various concentrations of recombinant DR0846. The reaction mixture was incubated at 42 °C for 15 min, and thermal aggregation of the substrate was estimated by monitoring the degree of turbidity at 340 nm using an Evolution 300 Spectrophotometer (Thermo Scientific) equipped with a thermostatic cell holder. The thermal aggregation of MDH was used as the control. The holdase chaperone activity of DR0846 was determined at 1 : 1, 1 : 2, and 1 : 3 molar ratios between substrate (MDH) and DR0846.

### AMS modification of DR0846 and DR0846 Cys mutant proteins

4‐Acetamido‐4′‐maleimidylstilbene‐2,2′‐disulfonic acid (AMS) modification was performed as described previously 34 with minor modifications. The proteins were precipitated by the addition of one volume of trichloroacetic acid (TCA), followed by incubation for 1 h at −20 °C. After 1 h, precipitates were collected by centrifugation at 18 400 g for 5 min and washed with ice‐cold acetone three times. Final pellets were dissolved in 20 μL of AMS working solution (50 mm Tris/HCl, pH 7.5, 0.1% SDS, 10 mm EDTA, 20 mm AMS) and incubated for 1 h at 25 °C in the dark. Samples were separated by reducing SDS/PAGE and analyzed by western blotting. For western blot, we generate antibody using purified DR0846 recombinant protein. DR0846 protein was immunize to mice and the antiserum was used for immunoblotting.

### Construction of deletion mutants in *D. radiodurans*

The ∆*dr0846* and ∆*dr1998* disruption mutants were constructed by targeted mutagenesis using the double cross‐over recombination method described previously 35, 36. Two amplified 1 kb fragments from upstream and downstream of the targeted genes were digested with appropriate restriction enzymes (Table S1), and ligated into the corresponding sites of kanamycin resistance cassette in pKatAPH3. The recombinant plasmids were transformed into *D. radiodurans* cells. The mutant strains were selected on TGY agar plates supplemented with 8 μg·mL^−1^ kanamycin. To construct the ∆*dr0846/*∆*dr1998* double‐deletion mutant*,* digested upstream and downstream fragments of *dr1998* gene were ligated into the corresponding sites of chloramphenicol resistance cassette in pKatCAT5. The recombinant plasmids were transformed into ∆*dr0846* mutant strain, and the transformant was screened on TGY agar plate containing kanamycin (8 μg·mL^−1^) and chloramphenicol (4 μg·mL^−1^). The deletions of genes were verified by diagnostic PCR and nucleotide sequencing.

### Hydrogen peroxide (H_2_O_2_) and heat stress tolerance assay

The sensitivity of *D. radiodurans* cells to hydrogen peroxide was assayed as described previously 37 with minor modifications. Cells were harvested in early stationary phase, washed twice and resuspended in phosphate buffer (20 mm, pH 7.4). The cells were serially diluted from 10^−1^ to 10^−4^. Cells were spotted on TGY plates containing 0 or 0.2 mm H_2_O_2_ and incubated at 30 °C for 16 h.

To study the heat resistance of *∆dr0846*, early stationary‐phase cells were used for the seed culture. The seed culture was inoculated in TGY broth at a 1 : 100 dilution. Cells were grown at 30 °C until OD_600_ of ~ 0.5 in TGY broth with or without 8 μg·mL^−1^ kanamycin. For thermal stress, cells were incubated at 30 °C or 42 °C for 30 min. The cells were serially diluted from 10^−1^ to 10^−4^. Diluted cells were spotted on TGY plates and incubated at 30 °C for 16 h.

## Results

### Phylogenetic analysis of peroxiredoxins from *D. radiodurans* R1

The peroxiredoxin (Prx) family is classified into four groups based on sequence properties: 1‐Cys Prx, 2‐Cys Prx, type II Prx, and PrxQ 38, 39. 1‐Cys Prxs contain only one conserved cysteine residue. 2‐Cys Prxs contain two conserved cysteine residues and both residues are essential for enzyme activity. Type II Prxs, also named atypical 2‐Cys Prxs, have two cysteine residues but the position of one of these cysteines is not conserved 38, 39. In addition, PrxQs contain two cysteine residues 38, 39.

The *D. radiodurans* R1 genome encodes four putative peroxiredoxins (DR0846, DR1208, DR1209, and DR2242) 3. To investigate the relationship between *D. radiodurans* Prxs and those of diverse organisms, a phylogenetic analysis was conducted using Prx family members from *Arabidopsis*, humans, yeast, *Synechocystis* sp., and *Chlamydomonas* (Fig. S1). The evolutionary history was inferred using the neighbor‐joining method 24, 26. All peroxiredoxins in *D. radiodurans* were assigned to the PrxQ, suggesting that PrxQs may have a vital role for the viability of *D. radiodurans* under oxidative stress.

### Expression analysis of peroxiredoxins in response to oxidative stress or gamma rays

**Figure 1:**
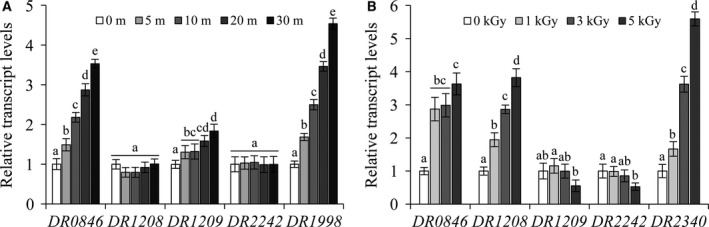
Expression analysis of Prx genes in response to oxidative stress or gamma rays. Cells were grown on TGY medium for 16 h at 30 °C. mRNA levels of peroxiredoxin genes were determined by qRT‐PCR after treatment with 20 mm H_2_O_2_ for 5–30 min (A) or treatment with gamma rays at 1–5 kGy (B). Data are means ± SE from three replications for each treatment. Different letters indicate significant differences at *P* < 0.05 between the groups by one‐way ANOVA with Tukey's test. *DR1343* (*gap*) was used as a loading control. *DR1998* (*KatE1*) and *DR2340* (*recA*) were used as positive controls for oxidative stress and gamma rays, respectively.

Peroxiredoxins are a family of antioxidant enzymes involved in sensing and detoxifying hydrogen peroxide (H_2_O_2_) and other ROS 40. To investigate the expression of *Deinococcus* peroxiredoxins in response to oxidative stress, we evaluated cells by qRT‐PCR after oxidative stress treatment. After 20 mm H_2_O_2_ treatment, *DR0846* and *DR1209* expression was induced, whereas *DR1208* and *DR2242* expression levels were not different from those in the control group (Fig. 1A). To test the expression of peroxiredoxin genes in response to gamma rays, we irradiated cells with 1, 3, and 5 kGy. The transcript expression levels of *DR0846* and *DR1208* increased gradually with increasing gamma irradiation, whereas *DR1209* and *DR2242* expression levels were unchanged until 3 kGy and decreased at higher doses (Fig. 1B). Based on these results, we selected PrxQ (*DR0846*), which is simultaneously induced by both H_2_O_2_ and gamma rays and showed a higher expression level than that of *DR1208*, for further analyses (Fig. 1B).

### Protein sequence and oligomeric state of DR0846

The sequence of DR0846 was compared with those of homologous PrxQ proteins from diverse organisms by a multiple sequence alignment. The conserved peroxidatic cysteine (*C*_p_) of DR0846 was located around position 60 in a PxxxTxxC‐motif (Fig. S2) 41. The sequence alignment also showed that there is an additional cysteine at position 65 in DR0846.

**Figure 2:**
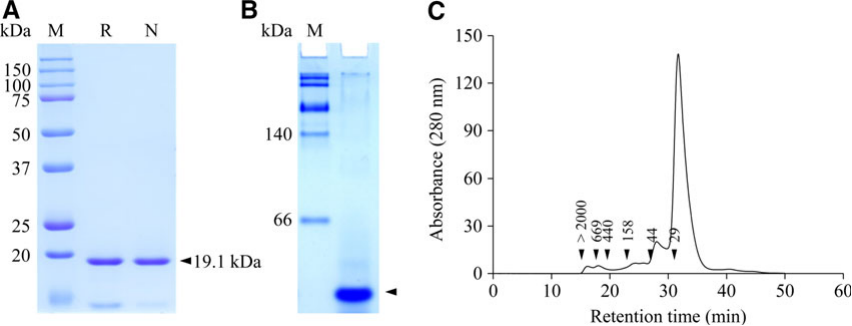
Purity and oligomeric state of DR0846 based on SDS/PAGE (A), native‐PAGE (B), and SEC (C). The proteins were denatured by heating in the presence (R) or absence (N) of 5 mm DTT. DR0846 was separated by 12% SDS/PAGE (A) or 10% native‐PAGE (B) and the gel was stained with Coomassie Blue. M, Marker; R, Reducing; N, Nonreducing. SEC was performed using a Superdex 200 10/300 column. The numbers in the chromatogram represent the molecular weights of the standard proteins; blue dextran (> 2000 kDa), thyroglobulin (669 kDa), ferritin (440 kDa), aldolase (158 kDa), ovalbumin (44 kDa), and carbonic anhydrase (29 kDa).

For biochemical studies of DR0846 encoding a protein of 175 amino acids with a theoretical molecular mass of 19.1 kDa, the recombinant protein was overexpressed in *E. coli* BL21 (DE3) and purified. The purified proteins were analyzed by SDS/PAGE (Fig. 2A) or native‐PAGE (Fig. 2B). As shown in Fig. 2A, the purified recombinant DR0846 protein showed a single band with a molecular mass of approximately 19 kDa by 12% SDS/PAGE in the presence (reducing) or absence (nonreducing) of DTT. To further determine the oligomeric status of the native DR0846 protein, we performed native‐PAGE and SEC (Fig. 2B,C). As shown in Fig. 2B,C, DR0846 existed primarily in monomeric form, with a small quantity of oligomeric structures without the formation of intermolecular disulfide bonds.

### Effect of cysteine residues on enzymatic activity of DR0846

**Figure 3:**
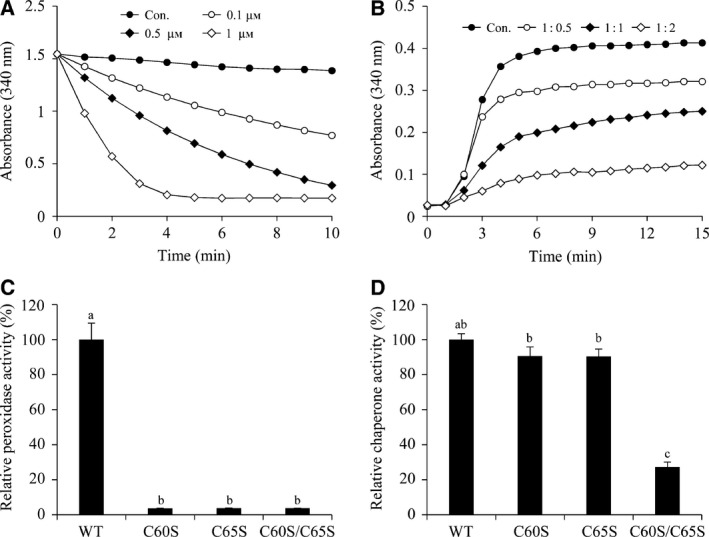
Peroxidase and chaperone activities of DR0846 and DR0846 Cys mutant proteins. (A) Peroxidase enzyme assay of DR0846. Peroxidase enzyme activity was measured using the yeast Trx system at various concentrations. (B) Molecular chaperone assay of DR0846. Chaperone activity was measured by the aggregation of MDH at 42 °C at different molar ratios of MDH/DR0846. Con., 1 : 0 (●); 1 : 0.5 DR0846 (○); 1 : 1 DR0846 (♦); 1 : 2 DR0846 (♢). Peroxidase enzyme assay (C) and chaperone enzyme assay (D) of DR0846 Cys mutant. C60S, C65S, and C60S/C65S are cysteine substitution mutants. Data are means ± SD of three independent experiments. Different letters indicate significant differences at *P* < 0.05 between the WT and mutant proteins by one‐way ANOVA with Tukey's test.

PrxQ proteins are thiol‐based peroxidase that catalyzes the reduction of hydrogen peroxide 19, 22. To investigate whether DR0846 possesses peroxidase activity, we conducted peroxidase activity assays (Fig. 3A). We measured the peroxidase enzymatic activity of DR0846 by monitoring the reduction of H_2_O_2_ by coupled NADH oxidation at 340 nm using the Trx (Trx, TR, and NADH) system. As shown in Fig. 3A, DR0846 showed peroxidase activity in a concentration‐dependent manner in the presence of the Trx system (Trx, TR, and NADH). Typical 2‐Cys Prx and some atypical 2‐Cys Prx‐type peroxidases use thioredoxin as a reductant. However, the donor substrate specificity of PrxQ remains unclear. It has been reported that *Burkholderia cenocepacia* BCP (BcBCP) uses thioredoxin as a reductant for the sulfenic acid intermediate 22. However, greater peroxidase activity is observed when using glutathione as an electron donor 22. Therefore, we assayed the efficiency of the GSH (GSH, GR, and NADH) systems in providing reducing power for DR0846 in the reduction of H_2_O_2_ (Fig. S3). No peroxidase activity was detected for DR0846 in the presence of the GSH system (Fig. S3). In contrast, erythrocyte GPx showed significant catalytic activity in the same system. These results indicated that DR0846 is a thioredoxin‐dependent PrxQ that uses thioredoxin as an electron donor and consistent with the fact that *D. radiodurans* has no GSH.

Recent studies have revealed that some peroxiredoxins have dual functions as a peroxidase and a chaperone 31, 42, 43. To investigate whether DR0846 act as a molecular chaperone, we performed a holdase chaperone activity assay using MDH as a heat‐sensitive substrate. The incubation MDH with increasing amounts of DR0846 prevented thermal aggregation of MDH at 42 °C, and aggregation was completely inhibited at a 1 : 2 molar ratio of MDH to DR0846 (Fig. 3B). These results indicate that DR0846 act as a peroxidase and a molecular chaperone.

Thiol peroxidase of *E. coli* has a functional cysteine residue that is a crucial for enzymatic activity 44. To investigate the physiological function of the cysteine residue on DR0846 enzymatic activity, we generated various substitution mutants of DR0846 by replacing cysteine residues with serine at the C60 and C65 positions. All of the mutants, i.e., C60S, C65S, and C60S/C65S, exhibited complete loss of peroxidase activity (Fig. 3C). The holdase chaperone activity of C60S and C65S single mutants was not altered, whereas the holdase chaperone activity of C60S/C65S was almost blocked (Fig. 3D). These results suggest that each Cys residues are important for peroxidase activity, whereas both Cys residues are required for molecular chaperone activity.

### Analysis of disulfide bond formation in DR0846

**Figure 4:**
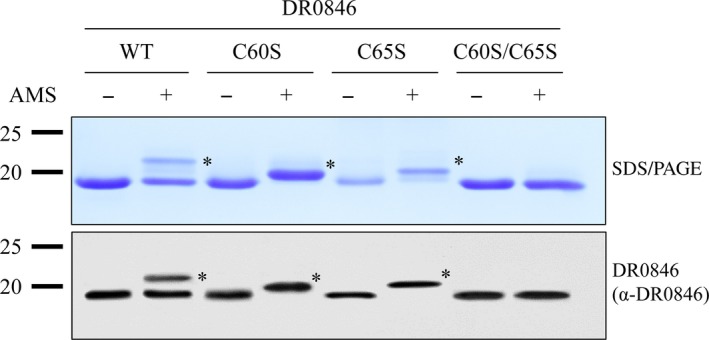
Redox properties of DR0846 and Cys mutant proteins. AMS shift assays were performed using purified WT DR0846 and cysteine mutant proteins. Purified proteins were precipitated with TCA, and treated without (−) or with (+) AMS. The proteins were resolved by reducing SDS/PAGE and subjected to a western blot analysis. Asterisks indicate the oxidized forms of each protein.

PrxQ possesses the PXXXXC motif, forms an intramolecular disulfide bond between two cysteine residues, of the catalytic site and adopts an atypical 2‐Cys catalytic mechanism 19. The amino acid sequence of DR0846 has only two cysteine residues (Fig. S2) and did not form an intermolecular disulfide bond (Fig. 2A). To test whether the cysteine in DR0846 forms an intramolecular disulfide bond, trapping experiments of free thiol (‐SH) groups were performed using wild‐type (WT) DR0846 and its Cys‐to‐Ser mutants (C60S, C65S, and C60S/C65S). The thiol alkylation agent AMS reacts with a free thiol (‐SH) group in the protein resulting from increasing the molecular mass by approximately 540 Da per AMS molecule. AMS‐modified and unmodified DR0846 were separated by reducing SDS/PAGE and detected by immunoblotting using DR0846 antibody (Fig. 4). In the presence of AMS, two bands were detected for WT DR0846 corresponding to the fully reduced form, showing a weight consistent with the binding of two AMS molecules (upper band) and oxidized form (lower band) with an intramolecular disulfide bond. Two forms of the protein were purified in atmospheric conditions, i.e., oxidized and reduced forms, but the oxidized form was slightly more highly represented. The C60S and C65S proteins exhibited only reduced forms showing a weight consistent with the binding of single AMS molecules (upper band). However, only one band, which was not modified by AMS, was observed for the C60S/C65S double mutant (Fig. 4). These results indicate that the two cysteines (C60 and C65) of DR0846 exhibit the free thiol groups or intramolecular disulfide bonds under redox status.

### Phenotypic characterization of the *dr0846* disruption mutant

**Figure 5:**
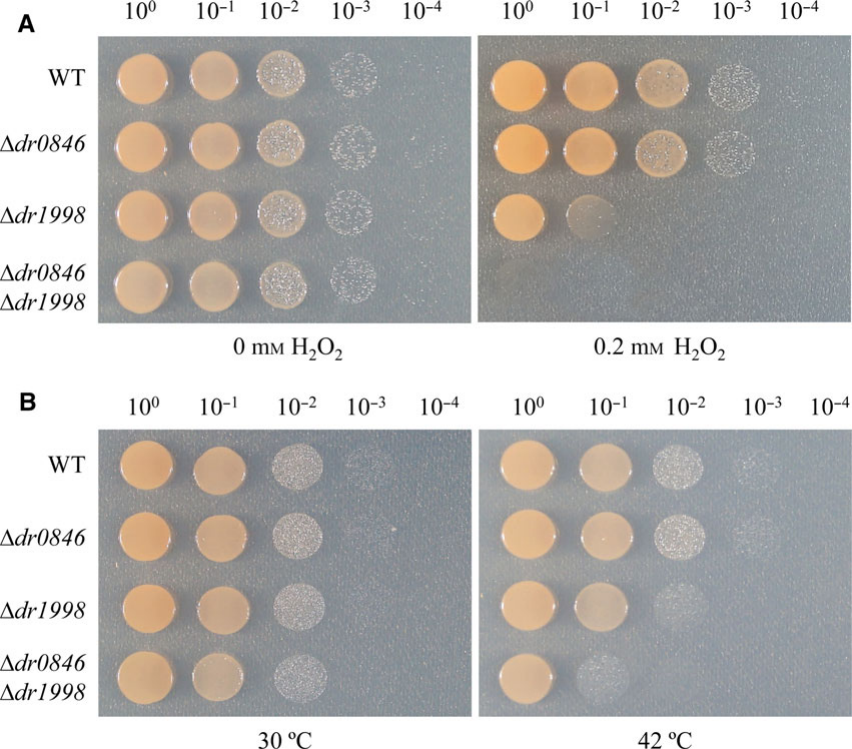
Hydrogen peroxide (H_2_O_2_) and heat stress tolerance assays of the ∆*dr0846* mutant. Exponential‐phase WT *Deinococcus radiodurans* and the deletion mutant (Δ*dr0846*) were grown on TGY plates containing different concentrations of H_2_O_2_ for 16 h (A) or incubated at 30 °C or 42 °C for 30 min and grown on TGY plates for 16 h, followed by serial dilution (B).

DR0846 showed peroxidase and molecular chaperone activities *in vitro* (Fig. 3A,B). To assess the role of DR0846 *in vivo*, we generated a *dr0846* disruption mutant and evaluated it by oxidative and heat stress tolerance assays (Fig. 5). However, as shown in Fig. 5, the sensitivity to H_2_O_2_ or heat stress of the *∆dr0846* mutant was similar to that of the WT. Catalases and peroxidases remove H_2_O_2_ and catalase activity is correlated with the lethal effects of heat shock stress 45. Catalase activities of exponential and stationary‐phase *D. radiodurans* were greater than those of *E. coli* 46. *D. radiodurans* possesses two catalases 3; among them, the typical monofunctional heme‐containing DR1998 is a major catalase 11. To rule out the possibility of DR1998 catalase function, we constructed catalase‐ and peroxidase‐deficient double‐mutant strains and conducted H_2_O_2_ and heat stress tolerance assays using log phase cells were subjected oxidative and heat stress tolerance assay. The ∆*dr1998* catalase single‐deletion mutants showed reduced growth compared with that of the WT after treatment with 0.2 mm H_2_O_2_ (Fig. 5A) or heat treatment (Fig. 5B). Furthermore, the ∆*dr0846* ∆*dr1998* double mutants showed severe growth retardation in response to H_2_O_2_ or heat stresses (Fig. 5). These results suggest that DR0846 has dual functions as a peroxidase and a molecular chaperone *in vivo*.

## Discussion

Peroxiredoxins catalyze the reduction of hydrogen peroxide and alkyl hydroperoxides 40. Although Prxs of various organisms have been studied, little is known about PrxQ *D. radiodurans* R1. These studies on *D. radiodurans* PrxQ, a member of peroxiredoxin, provide a new insight into the structure and function of peroxiredoxin. Here, we reports the substrate and donor specificity and *in vivo* function of DR0846 from *D. radiodurans*. As expected, *D. radiodurans* PrxQ acts as a thioredoxin‐dependent monomeric peroxidase and molecular chaperone, which has two cysteine residues important for its activity.

The typical 2‐Cys Prx have been reported to have physiological functions as peroxidase and molecular chaperone 28, 29, 33, 42, 47, 48. Ionic interactions play an important role in the oligomerization and function of Prx proteins 49, 50. The typical 2‐Cys Prx dynamically regulates the structure between low molecular weight and high molecular weight (HMW) in response to cellular redox states and this structural change is important for the switch between peroxidase and chaperone function 28, 29, 31, 42, 48. 1‐Cys Prx, PrxQ, and Type II Prx act as monomers, whereas 2‐Cys Prxs act as dimers. However, the oligomeric complex has been detected for 1‐Cys Prx and 2‐Cys Prx, whereas PrxQ and Type II Prx have been observed as monomers or dimers 47, 50, 51. The *Corynebacterium glutamicum* PrxQ (CgPrxQ) and *E. coli* BCP are predominantly present as monomers in the native or functional states 19, 52. PrxQ4 (ScBCP4) forms a dimer with a non‐covalent dimeric structure 20.

Molecular chaperones prevent the aggregation and assist the covalent folding of proteins and oligomeric architectures are important for chaperone functions 53, 54. While most typical 2‐Cys Prx, some 1‐Cys Prx and CgPrxQ have been reported to have peroxidase activity and oligomeric or dimeric chaperone activity 28, 29, 31, 33, 42, 55, 56, this is the first report for the dual activity of a monomeric atypical 2‐Cys Prx. Although it is not known in Prx, this observation is similar to the results of other proteins reported previously. The FanE is a monomeric chaperone that is present in the periplasm of *E. coli* 57. Trigger Factor (TF) from *Psychrobacter frigidicola* (TF_*Pf*_) displays no dimerization and it can promote refold of RNase T1 58. The monomeric 14‐3‐3ζ protein has a chaperone‐like activity and the 14‐3‐3ζ more effectively prevents aggregation of myosin subfragment 1 compared with its dimeric form. In addition, HSP27, most abundant small heat shock protein in humans, the reduced form was more effective than its oxidized form in preventing protein aggregation 59. However, the molecular mechanism underlying the chaperone activity of monomeric proteins needs to be further studied.

Recent studies revealed that the resistance of *D. radiodurans* to variety of stresses conditions can be explained by high antioxidant activity to protect cells 1, 3, 5, 10, 11. Heat stress causes the production of ROS to induce oxidative stress and affects antioxidant enzyme activities such as catalase, SOD, and peroxidase in plant species 60, 61. In plants, catalase activities and intensities of catalase isozymes may be important determinants of antioxidant resistance to heat stress 62. The peroxidase enzyme has been associated with the emergence of physiological injuries and its activity was enhanced by high‐temperature stress in mulberry and strawberry 63, 64. Despite previous studies of antioxidant enzymes in relation to heat tolerance, specific antioxidant enzymes have not been well‐characterized. In this study, DR0846 showed strong peroxidase and chaperone activity and exhibited a sensitive phenotype for heat stress based on a mutation analysis (Figs 2 and 4). These results suggest that PrxQ (DR0846) may be an important antioxidant enzyme involved in thermal stress resistance.

## Author contributions

CC and SSL conceived and designed the study; CC, GWL, SHH, SK, and JJ performed the experiments; CC, KJ, JJ, SL, BYC, and SSL analyzed the data; CC, SL, and SSL wrote the manuscript.

## Supporting information


**Fig. S1.** Phylogenetic analysis of peroxiredoxins in *D. radiodurans* R1 and diverse organisms.
**Fig. S2.** Alignment and sequence comparison of DR0846 with PrxQ proteins from diverse organisms.
**Fig. S3.** GSH‐dependent peroxidase activity of DR0846 under GSH system.
**Table S1.** List of primers used in this study.
