# Supplementary material for: Novel functions of peroxiredoxin Q from Deinococcus radiodurans R1 as a peroxidase and a molecular chaperone
Source: FEBS Lett. 2018 Dec 11;593(2):219–29. doi: 10.1002/1873-3468.13302 (PMC6590489; doi:10.1002/1873-3468.13302)
Supplement: Supplementary file 1 — Fig. S1. Phylogenetic analysis of peroxiredoxins in D. radiodurans R1 and diverse organisms. Fig. S2. Alignment and sequence comparison of DR0846 with PrxQ proteins from diverse organisms. Fig. S3. GSH‐dependent peroxidase activity of DR0846 under GSH system. Table S1. List of primers used in this study. [file FEB2-593-219-s001.docx]

**
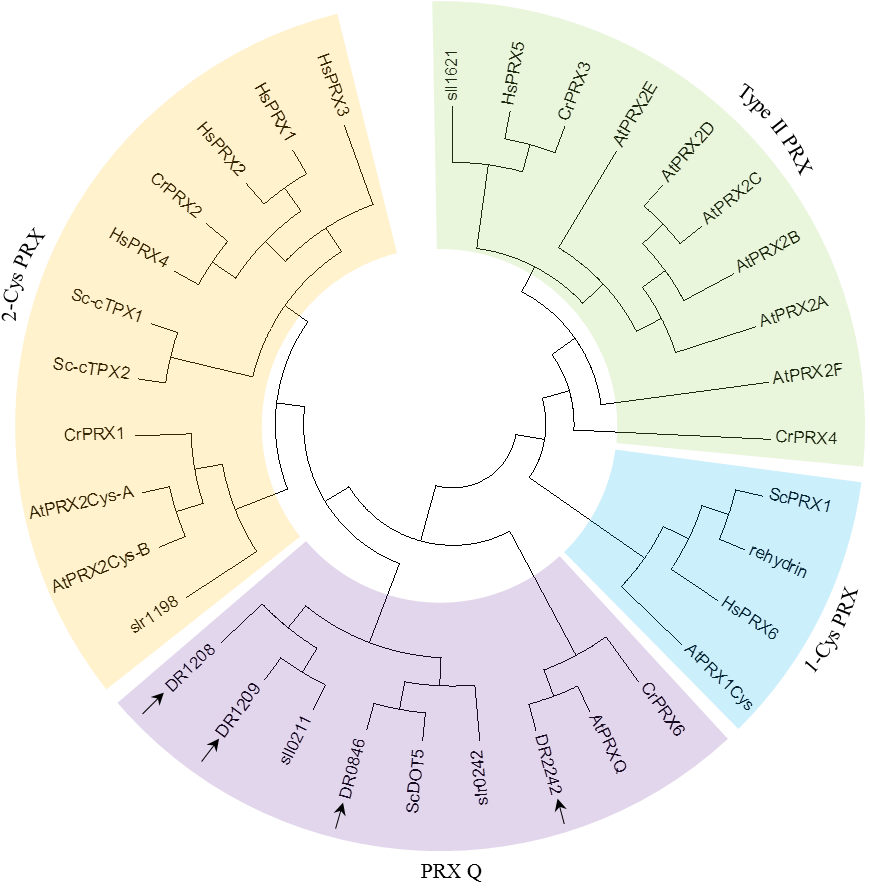
**

**Supplementary Fig. S1.** Phylogenetic analysis of peroxiredoxins in *D. radiodurans* R1 and diverse taxa. The sequences were aligned and compared to construct an unrooted phylogenetic tree using MEGA7. Arrows indicate PRX of *D. radiodurans* R1. Accession numbers and abbreviations are as follows: At, *Arabidopsis thaliana*; AtPRX2A, At1g65990; AtPRX2B, At1g65980; AtPRX2C, At1g65970; AtPRX2D, At1g60740; AtPRX2E, At3g52960; AtPRX2F, At3g06050; AtPRX2Cys-A, At3g11630; AtPRX2Cys-B, At5g06290; AtPRX1Cys, At1g48130; and AtPRXQ, At3g26060; Cr, *Chlamydomonas reinhardtii*; CrPRX1, EDP08588.1; CrPRX2, EDP07356.1; CrPRX3, EDP07991; CrPRX4, EDP06862; CrPRX6, EDP06371; DR, *Deinococcus radiodurans*; *R1* DR0846, NP294570.1; DR1208, NP294932.1; DR1209, NP294933.1; DR2242, NP295964.1; Hs, *Homo sapiens*; HsPRX1, Q06830; HsPRX2, P32119; HsPRX3, P30048; HsPRX4, Q13162; HsPRX5, P30044; HsPRX6, P30041; Sc, *Saccharomyces cerevisiae*; Sc-cTPX1, P34760; Sc-cTPX2, Q04120; ScPRX1, P34227; ScDot5, P40553; sll or slr, *Synechocystis PCC6803*; rehydrin, BAK49553; sll0221, BAL34236; sll1621, P73728; slr0242, BAL34214; and slr1198, BAL34890.

▼

▼

**Supplementary Fig. S2.** Alignment and sequence comparison of DR0846 with PrxQ proteins from diverse organisms. The alignment was obtained using ClustalW (version 1.7). Identical amino acid residues are highlighted in black and residues shaded in gray are similar. The arrowhead indicates the two conserved cysteine residues in DR0846.

**
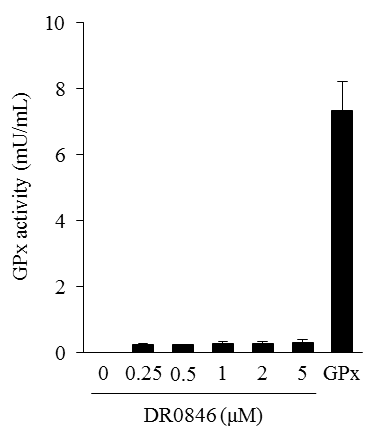
**

**Supplementary Fig. S3.** GSH-dependent peroxidase activity of DR0846 under GSH system. The enzymatic activity was measured using various concentrations of DR0846. Error bars represent means ± SD of three independent experiments.

| **Supplementary Table S1.** List of primers used in this study   \| Name \| Sequence (5' to 3')^a^ \| Description \| \| --- \| --- \| --- \| \| DR0846 qRT-F \| 5'-ACCAGGGAAGCCTGTGATTT-3' \| qRT-PCR \| \| DR0846 qRT-R \| 5'-TACTTGTCGGCAAACTGCTG-3' \| qRT-PCR \| \| DR1208 qRT-F \| 5'-GCCCGCGTTCAAATTGCC-3' \| qRT-PCR \| \| DR1208 qRT-R \| 5'-GCGAATGAGCGAGTGGATAA-3' \| qRT-PCR \| \| DR1209 qRT-F \| 5'-CCGAGGCTTATGGAGTGCT-3' \| qRT-PCR \| \| DR1209 qRT-R \| 5'-GGTTAACGCGTTTCCAGTGA-3' \| qRT-PCR \| \| DR2242 qRT-F \| 5'-ATCACCCTCAGCAGCTACC-3' \| qRT-PCR \| \| DR2242 qRT-R \| 5'-TTGATGCCGAGCACAACG-3' \| qRT-PCR \| \| KatE1 qRT-F \| 5'-ACCTTCAGCTACTCCGACAC-3' \| qRT-PCR \| \| KatE1 qRT-R \| 5'-GTCCTGGCCCTCAAAGGTAT-3' \| qRT-PCR \| \| recA qRT-F \| 5'-CCGCCATCTTCATCAACCAG-3' \| qRT-PCR \| \| recA qRT-R \| 5'-TTGATCTTGACGGTGTTGGC-3' \| qRT-PCR \| \| gap qRT-F \| 5'-ATCAACGACCTGACCGACAA-3' \| qRT-PCR \| \| gap qRT-R \| 5'-CGGTGGATTCGATCACGATG-3' \| qRT-PCR \| \| DR0846 *Bam*HI \| 5'-CGCGGATCCATGACCGATTCTGATTCCCAGAGCC-3' \| Protein expression \| \| DR084*6 Hin*dIII \| 5'-CCCAAGCTTTCAGGCCTGGCCCTTCTTTTGGAG-3' \| Protein expression \| \| DR0845 *Xba*I F \| 5'-TGCTCTAGAATGGACCTTGAAGCCCTGAAAAAAGAAGC-3' \| *dr0846* deletion mutant \| \| DR0845 *Pst*I R \| 5'-AAAACTGCAGCCAATGATAATCAGCCGCTTCGCCTGCAC-3' \| *dr0846* deletion mutant \| \| DR0847 *Kpn*I F \| 5'-CGGGGTACCATGGTCGGCATCCGATTTACCCACAT-3' \| *dr0846* deletion mutant \| \| DR0847 *Eco*RV F \| 5'-CCGGATATCTCAGGACTTGGGATACAGCACGGC-3' \| *dr0846* deletion mutant \| \| dr1998_up_F *Xho* I \| 5'-ATTCTCGAGTCCTTGACCAGCCGGGTGCA-3' \| *dr1998* deletion mutant \| \| dr1998_up_R *Eco*RV \| 5'-TTAGATATCACACTCTCCTTCGCCTCGCTGGCT-3' \| *dr1998* deletion mutant \| \| dr1998_dw_F *Xba* I \| 5'-ATTTCTAGAGACAAGCTGAGCACGGA-3' \| *dr1998* deletion mutant \| \| dr1998_dw_R *Pst* I \| 5'-TTACTGCAGGCTGCGGATTTTCATAGAGGTA-3' \| *dr1998* deletion mutant \| \| DR0846 C60 F1 \| 5'-ATGACCGATTCTGATTCCCAGAGCC-3' \| Site-directed mutagenesis \| \| DR0846 C60 R1 \| 5'-ACAGGCTTCCCTGGTGGAGCC-3' \| Site-directed mutagenesis \| \| DR0846 C60 F2 \| 5'-GGCTCCACCAGGGAAGCCTGT-3' \| Site-directed mutagenesis \| \| DR0846 C60 R2 \| 5'-TCAGGCCTGGCCCTTCTTTTGCA-3' \| Site-directed mutagenesis \| \| DR0846 C65 R1 \| 5'-GCGGAAATCAGAGGCTTCCCTGGT-3' \| Site-directed mutagenesis \| \| DR0846 C65 R2 \| 5'-ACCAGGGAAGCCTCTGATTTCCGC-3' \| Site-directed mutagenesis \|   ^a^ Tags with restriction sites are underlined |  |
| --- | --- | --- | --- | --- | --- | --- | --- | --- | --- | --- | --- | --- | --- | --- | --- | --- | --- | --- | --- | --- | --- | --- | --- | --- | --- | --- | --- | --- | --- | --- | --- | --- | --- | --- | --- | --- | --- | --- | --- | --- | --- | --- | --- | --- | --- | --- | --- | --- | --- | --- | --- | --- | --- | --- | --- | --- | --- | --- | --- | --- | --- | --- | --- | --- | --- | --- | --- | --- | --- | --- | --- | --- | --- | --- | --- | --- | --- | --- | --- | --- | --- | --- | --- | --- | --- | --- | --- | --- | --- | --- | --- | --- | --- | --- |
